# Pathophysiologic Role of Neurotransmitters in Digestive Diseases

**DOI:** 10.3389/fphys.2021.567650

**Published:** 2021-06-14

**Authors:** Xiaoxu Yang, Jun Lou, Weixi Shan, Jianhong Ding, Zhe Jin, Yanxia Hu, Qian Du, Qiushi Liao, Rui Xie, Jingyu Xu

**Affiliations:** Department of Gastroenterology, Affiliated Hospital of Zunyi Medical University, Zunyi, China

**Keywords:** neurotransmitters, gastrointestinal inflammation, liver fibrosis, dopamine, serotonin

## Abstract

Neurotransmitters are special molecules that serve as messengers in chemical synapses between neurons, cells, or receptors, including catecholamines, serotonin, dopamine, and other neurotransmitters, which play an important role in both human physiology and pathology. Compelling evidence has indicated that neurotransmitters have an important physiological role in various digestive diseases. They act as ligands in combination with central or peripheral receptors, and transmits signals through chemical synapses, which are involved in regulating the physiological and pathological processes of the digestive tract organs. For instance, neurotransmitters regulate blood circulation and affect intestinal movement, nutrient absorption, the gastrointestinal innate immune system, and the microbiome. In this review, we will focus on the role of neurotransmitters in the pathogenesis of digestive tract diseases to provide novel therapeutic targets for new drug development in digestive diseases.

## Introduction

Neurotransmitters are not only hormonal factors, but are also a cell signaling factors. They are specific chemical substances that act as “messengers” in nervous and synaptic transmission. As ligands, they exert function via binding to their corresponding receptors on the plasma membrane of peripheral and central cells. At present, neurotransmitters are primarily divided into four categories: biogenic amines, amino acids, peptides, and others. Biogenic amines include serotonin (5-HT), dopamine (DA), norepinephrine (NE), and epinephrine (E). Amino acid neurotransmitters include: gamma-aminobutyric acid (GABA), glycine, glutamate, histamine, and acetylcholine (Ach). Peptide neurotransmitters are classified into: endogenous opioid peptide, substance P, neurotensin, cholecystokinin, somatostatin, vasopressin, oxytocin, and neuropeptide y. Other neurotransmitters are classified into nucleotides, arachidonic acid, and the like. Numerous studies have confirmed that common neurotransmitters are involved in regulating multiple systems, including cardiovascular, nervous, respiratory, digestive, and immune. For example, norepinephrine, adrenaline, etc. regulate myocardial contraction and control coronary artery contraction and relaxation (Daly and Sole, 1990), and dopamine deficiency is a key change in Parkinson’s disease (Segura-Aguilar et al., 2014). In this review, we summarize currently available information on the effect of neurotransmitters in digestive diseases, and discuss the probable molecular mechanisms of neurotransmitters in the pathogenesis of these diseases (Table 1). Improved knowledge of these mechanisms should help in designing targeted therapies able to halt or reverse disease progression.

**TABLE 1:** A summary of roles of neurotransmitters receptors in the pathological mechanism of digestive diseases.

Neurotransmitters receptors	Target cells	Related diseases	Biological effect	Authors/References
**5-HT**				
5- -HT1D, 5-HT2B	HCC cells	HCC	• Promotes the viability and proliferation of HCC • Promotes hepatocarcinogenesis	Liang et al., 2013; Niture et al., 2018; Zuo et al., 2019
5-HT2A, 5-HT2B	HSCs	Hepatic fibrosis	• Promotes HSCs proliferation, transcription	Ruddell et al., 2006; Ebrahimkhani et al., 2011; Kim D. C. et al., 2013
5-HT3		IBS	• Relieves abdominal pain, inhibits hypermotility	Salaga et al., 2018
5-HT4	Esophage	Reflux esophagitis and non-erosive reflux disease	• Relates with the contraction of the lower esophageal muscle	Yang et al., 2012
	Colonic epithelium	IBD	• Maintains motility • Reduces inflammation	Spohn et al., 2016
		IBS	• Inhibits visceral hypersensitivity	Hoffman et al., 2012; Gilet et al., 2014
5-HT7	GI epithelial cells	Infective acute enteritis, colitis, IBD	• Pro-inflammation	Kim J. J. et al., 2013
			• Anti-inflammation	Guseva et al., 2014
**Catecholamines**				
DRD1	Gastrointestinal mucosa	Stress-induced gastric ulcers	• Reduces the incidence of gastric and duodenal ulcers	Rasheed et al., 2010
	iNKT cells	Autoimmune hepatitis	• Suppress iNKT cell-mediated hepatitis	Xue et al., 2018
DRD2	Pancreatic acinar cells	AP	• Controls inflammation. • Reduces pancreatic damage	Han et al., 2017, 2020
	Pancreatic ductal adenocarcinoma cells	Pancreatic ductal adenocarcinoma	• Promotes proliferation of pancreatic cancer cells	Jandaghi et al., 2016
	Gastric tumor endothelial cells	GC	• Suppresses gastric cancer cell proliferation, invasion and migration	Chakroborty et al., 2004; Ganguly et al., 2010; Huang et al., 2016
	HCC cells	HCC	• Suppresses liver cancer cells proliferation migration and invasion • Reduces EMT, inhibits liver tumor growth	Li et al., 2015; Liu et al., 2017
ADRB2	BM-DMs	Colitis	• Suppress inflammation	Agac et al., 2018
	Gastric cancer cells	GC	• Promotes gastric cancer progression, metastasis, angiogenesis • Induces autophagy	Lu et al., 2017; Zhang et al., 2019; Zhi et al., 2019
	Pancreatic cancer cells	Pancreatic cancer	• Accelerates pancreatic cancer growth and invasion • Promotes angiogenesis and metastasis of pancreatic cancer	Hu et al., 2010; Kim-Fuchs et al., 2014
	HCC cells	HCC	l lPromotes HCC progression	Wu et al., 2016
ADRA1	HSCs	Hepatic fibrosis	• Promotes HSCs activation, proliferation and secretion of ECM	Sancho-Bru et al., 2006; Liu et al., 2014
	KCs, HCC cells	HCC	• Boosts the activation of KCs and to maintain the inflammatory microenvironment	Han et al., 2008; Huan et al., 2017
ADRA2	HCC cells	Hepatocellular dysfunction in early sepsis	• Induces hepatocellular dysfunction	Yang et al., 2001
**Glutamate receptors**				
**iGluR**				
AMPA	Colon endothelial cells	Colitis	• Enhance the efficiency of peristalsis	Giaroni et al., 2000
	Pancreatic cancer cells	Pancreatic cancer	• Increased invasion and migration	Herner et al., 2011
NMDA	Colon endothelial cells	Ulcerative colitis	• Promoted colon motility and inflammation	Erces et al., 2012; Motaghi et al., 2016
	Colon endothelial cells	GI diseases	• Induced proinflammatory neuropeptides, calcitonin gene-related peptide and substance • Increased colonic anaphylaxis	Cao et al., 2008; Fan et al., 2009
	Gastric epithelial cell	GC	• Resulted in Ca^2+^ permeation and epithelial cell death	Seo et al., 2011
	Colon adenocarcinoma cells	Colon adenocarcinoma	• Limited tumor growth	Rzeski et al., 2001
	Kupffer cells	Hepatitis	• Limited inflammasome and injury	Farooq et al., 2014
		Pancreatic neuroendocrine tumor	• Controlled invasion of tumor	
**mGluR**				
mGluR5	Esophage epithelial cell	GERD	• Triggered TLESRs and gastroesophageal reflux	Frisby et al., 2005
		IBS, FD	• Promoted visceromotor and autonomic responses	Lindstrom et al., 2008
	HSCs	Hepatic fibrosis	• Stimulated 2-AG production	Choi et al., 2019
mGluR7	Colon mucosa	GI dysfunction	• Attenuated visceral hypersensitivity	Shao et al., 2019

## 5-HT and Its Receptor

Serotonin, also known as 5-hydroxytryptamine or 5-HT, is produced in the central nervous system and in enterochromaffin cells (EC) of the gastrointestinal tract, playing an important role in the human body as an intermediate messenger (Mawe and Hoffman, 2013). Ninety percent of 5-HT in the body is synthesized and secreted by EC cells in the intestine, while only a small part is synthesized by neurons. The serotonins secreted by EC cells primarily acts in a paracrine manner (Bertrand, 2004).

Production of 5-HT is first generated by tryptophan under the action of tryptophan hydroxylase (TPH) to produce 5-hydroxytryptophan, which then, under the action of 5-hydroxytryptophan decarboxylase, produces 5-HT, which is stored in EC cell vesicles. 5-HT binds to 5-HT receptors, dissociating rapidly, and the dissociated 5-HT is actively absorbed by cells expressing Na^+^/Cl^–^ dependent serotonin transporter (SERT). It is stored in intracellular vesicles and released in response to exposure to various stimuli (Mawe and Hoffman, 2013). Less than 1% of 5-HT circulates in the blood in its free state, leaving the rest stored in platelets and presynaptic neurons (Da Prada and Picotti, 1979).

In the periphery, 5-HT mediates many physiological processes, such as vasoconstriction, vasodilation, gastrointestinal motility, cell proliferation, apoptosis, and platelet aggregation (Mammadova-Bach et al., 2018). Interestingly, platelets express SERT but do not express TPH, so they do not produce 5-HT but can take up intestinal 5-HT during intestinal circulation and carry 5-HT into the blood circulation (George, 2000). 5-HT functions largely as a ligand by binding to 5-HT receptors, which are widely located in both central and peripheral regions. The wide distribution of 5-HT receptors facilitates diverse biological effects, and at least 7 major classes of human 5-HT receptors have been currently identified, denoted as 5-HT1-7 (Shajib and Khan, 2015). 5-HT1 has five receptor subclasses, 5-HT1A, 5-HT1B, 5-HT1D, 5-HT1E, and 5-HT1F, 5-HT2 has three subclasses, 5-HT2A, 5-HT2B and 5-HT2C, 5-HT5 have two subclasses, 5-HT5A and 5-HT5B (Hannon and Hoyer, 2008). Except for the 5-HT3 receptor (the receptor is a gated Na+/K+ channel), all members of the serotonin receptor family belong to G protein-coupled receptors (Connolly and Wafford, 2004). At present, it is believed that 5-HT1, 5-HT2, 5-HT3, 5-HT4, and 5-HT7 are the primary serotonin receptors affecting gastrointestinal function (Shajib et al., 2017).

Previous studies have shown that there are numerous clinical diseases involving 5-HT signaling, including migraine depression, cardiovascular disease, schizophrenia, Alzheimer’s disease and so on (Nishio et al., 2003; Ferrero et al., 2017; Kraus et al., 2017; Shah and Gonzalez-Maeso, 2019). However, with further studies, the focus of 5-HT’s effects on the nervous system have now turned to their physiological and pathological effects in digestive disease.

### 5-HT Deficiency Contributes to Esophageal Motility Disorder

5-HT is closely related to gastrointestinal motility and plays a major role in the pathogenesis of gastro-esophageal acid reflux disease (GERD). Shiina et al. (2016) found that serotonin induces the contractile response of longitudinal smooth muscle in the mucosa of the esophageal muscle layer, and this process is mediated through activation of serotonin 5-HT 1 and 5-HT 2 receptors on muscle cells. Yang et al. (2012) compared biopsies of patients with reflux esophagitis and non-erosive reflux disease and found that 5-HT was significantly elevated in the former lesions, while the latter showed significantly reduced expression of SERT mRNA and 5-HT4 receptors. Furthermore, Saegusa et al. (2011) found that inhibition of 5-HT4 receptor activity weaken the contraction of the lower esophageal and cause reflux. Therefore, the relationship between esophageal disease and serotonin is one that is primarily centered on the use of serotonin reuptake inhibitors (SRIs) and serotonin agonists for treatment. Although SRIs and serotonin agonists are only sparsely used in the management of upper gastrointestinal (GI) tract disorders, studies are looking into their use to treat esophageal motility disorders (Karamanolis et al., 2015; Scheerens et al., 2015), and hypersensitive esophagus (Viazis et al., 2012).

### The Role of 5-HT in Gastrointestinal Diseases

Serotonin is an essential gastrointestinal signaling molecule, whose signaling plays a critical role in the pathophysiological mechanisms of gastrointestinal diseases. Serotonin is related to gastrointestinal visceral hypersensitivity (Grundy, 2008), and inflammatory responses (Mayer, 2011; Drossman, 2016). The visceral showed exhibits high sensitivity to increased plasma 5-HT levels (Kerckhoffs et al., 2012). Studies demonstrated that visceral pain relief is primarily related to 5-HT4 receptor. Hoffman et al. (2012) suggested that the activation of colonic mucosal 5-HT4 receptors accelerates propulsive motility and inhibit visceral hypersensitivity. YKP10811, a new and potent 5-HT4 receptor partial agonist, attenuate acute colonic hypersensitivity (Gilet et al., 2014). It recently became known that the 5-HT receptor 3 agonist relieves abdominal pain in a mouse model of irritable bowel syndrome (Salaga et al., 2018). Stress-induced visceral hyperalgesia is abolished in a model of stress-induced sensitization of visceral nociception in rats by using the 5-HT3 receptor antagonist alosetron (Rapalli et al., 2016). All findings showed that 5-HT and activation of 5-HT3 and 4 receptors inhibit visceral sensitivity and relieve pain. However, while this protective phenomenon has been clearly documented, the mechanism of action of these compounds has not been clearly resolved.

Furthermore, 5-HT regulates inflammation by affecting the immune system (Hanoun et al., 2015; Yoo and Mazmanian, 2017). Rather than a comprehensive examination of the pro- and anti-inflammatory activities of 5-HT in the gut, here, we focus on the bidirectional neuroimmune interactions in the regulation and consequences of intestinal inflammation, as well as the central roles that serotonin plays as a signaling molecule in triggering, enhancing, and countering inflammation. Many different types of immune cells, including T cells, macrophages, mast cells, dendritic cells and platelets, express the machinery to generate, store, respond to and transport serotonin (Wu et al., 2019). Wang et al. (2013) found that the 5-HT1A receptor is primarily expressed in the enteric nervous system, particularly in the submucosa and intestinal myenteric plexus, regulating degranulation of mast cells and release of mediators. In various animal experiments, serotonin’s role in the gastrointestinal inflammatory response has been clarified. It was reported that secretion of cellular inflammatory factor is significantly reduced in dendritic cells of TPH1 knockout mice with colitis, and T cells induced by dendritic cells reduce levels of pro-inflammatory cytokines IL-17 and interferon-γ in TPH1 knockout mice (Li et al., 2011). Subsequent work established that the severity of inflammation is significantly diminished in mice lacking TPH1 owing to the selective ablation of mucosal 5-HT (Ghia et al., 2009), and when immunodeficient mice are reconstituted with effector T cells, the number of EC cells and the levels of 5-HT were significantly increased (Motomura et al., 2008). These reports substantiate the neuroimmune interactions in the gut.

In a mouse model of dextran sulfate sodium-induced colitis established by Chen’ team, 5-HT has been discovered to exacerbate colitis (Chen et al., 2016). This interesting phenomenon does not occur by accident. In the latest article from Shajib’s group, they found that EC-derived mucosal 5-HT acts as a pro-inflammatory mediator by regulating activation of immunocytes in intestinal inflammation, resulting in increased proinflammatory cytokines and decreased mucin production (Shajib et al., 2017). They demonstrated that 5-HT released from EC cells enhances inflammation through its action on 5-HT7 receptors, which are expressed by dendritic cells. Unfortunately, as compelling as this idea seems to be, strong evidence has been advanced for an equally compelling but conflicting hypothesis. Contrary evidence suggests that the dendritic cell 5-HT7 receptor is anti-inflammatory, not pro-inflammatory. A 5-HT7 antagonist, SB-269970, and deletion of 5-HT7 receptors are found to increase the severity of inflammation, and stimulation of the 5-HT7 receptor exerted anti-inflammatory effects (Guseva et al., 2014). A difference between the studies is that the proinflammatory side (Kim J. J. et al., 2013) employed a dose of SB-269970 that is 2500-fold higher than that utilized by the anti-inflammatory advocates (Guseva et al., 2014). Clearly, 5-HT from EC cells cannot drive inflammation through the 5-HT7 receptors of dendritic cells if stimulation of these receptors opposes inflammation. Therefore, the pro-inflammatory response to EC cells by 5-HT remains to be clarified. This interesting phenomenon has proven not to be accidental in individual receptors. Subsequent study has shown that blocking the 5-HT1A receptor also increases the severity of colitis induced by 2,4,6-trinitrobenzene sulfonic acid (Rapalli et al., 2016). Animal research by Spohn et al. (2016) found that activation of 5-HT4R maintains motility of healthy colons in mice and guinea pigs, reducing inflammation in the colons of mice with colitis, and exerting protective effects on normal and inflamed colon. The presence of anti-inflammatory targets in the intestinal lining makes the development and testing of restricted 5-HT4 agonists an interesting opportunity for potentially safe and effective treatment of inflammatory bowel disease (IBD).

Clinical research on the pro-inflammatory effects of 5-HT are not yet entirely clear. Long-term mental stimulation cause gut brain axis dysfunction, giving rise to an increase in the number of ECs in the intestinal mucosa, and serum serotonin levels have been found to increase, as well (El-Salhy et al., 2013). Interleukin and bacterial lipopolysaccharide are also observed to stimulate increased 5HT secretion from EC cells which isolated from the mucosa of individuals with Crohn’s disease compared to those of control subjects (Kidd et al., 2009). In humans, preoperative administration of a 5-HT4 receptor agonist, prucalopride, decreases IL6 and IL8 expression in the muscularis external and improved clinical recovery (Stakenborg et al., 2019). The effects of mechanical forces and adenosine receptors that drive 5-HT secretion from EC cells have also been found to be amplified in IBD (Chin et al., 2012). These observations illustrate the powerful effects, for better or worse, that altered neuronal function exerts on the structure of the gut and its subsequent behavior. They also suggest the promising, yet not fully exploited, therapeutic potential of neuroactive compounds.

**FIGURE 1:**
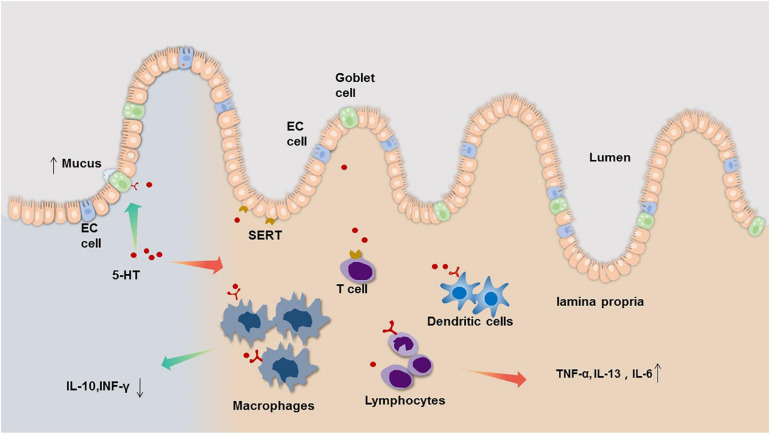
The bidirectional neuroimmune interactions of 5-HT in the regulation and consequences of intestinal inflammation. Evidence from clinical and animal studies indicate that EC cells of the gastrointestinal (GI) tract are the main source of mucosal 5-HT, which acts a pro-inflammatory mediator by regulating immune cell activation leading to increased pro-inflammatory cytokine, such as TNF-α, IL-13, IL-6 **(right side)**. However, 5-HT also can act on 5-HT receptors on goblet cells to increase mucus secretion, and decrease IL-10, INF-γ through regulating 5-HT receptors on immune cell **(left side)**.

Therefore, we infer that peripheral serotonin signaling has both pro-inflammatory and anti-inflammatory effects in the intestinal tract, acting as both a ‘sword and a shield.’ Under normal physiological conditions, these two effects exist in equilibrium, but once the balance is broken, 5-HT can become both a pro-pathological factor and a potentially protective factor (Figure 1). The gastrointestinal tract is a complex system that is intricately controlled by several modulators. Local mediators, central nervous system, enteric nervous system as well as hormones produced by other organs all influence 5-HT concentrations and its end effect on gut physiology. The mechanism involved in these processes is not clear and needed to explore.

### The Function of 5-HT in Liver Diseases

Hepatic fibrosis is a wound healing response to a variety of chronic stimuli, characterized by excessive deposition of extracellular matrix (ECM) proteins. Activated hepatic stellate cells (HSCs) are responsible for excess collagen deposition during liver fibrosis (Parsons et al., 2007). They lose their characteristic lipid droplets and are “activated” during liver injury. Previous studies have demonstrated that HSCs primarily express 5-HT2A, and 2B receptors and HSCs uptake and release 5-HT through the 5-HT receptor (Ruddell et al., 2006). The effect of 5-HT on hepatic fibrosis may occur by affecting activation of HSCs through the 5-HT receptor signaling pathway of HSCs. This hypothesis is confirmed in multiple studies on the relationship between 5-HT and HSCs activation. 5-HT may act a pre-fibrotic factor in the diseased liver. 5-HT2A, and 2B receptors mediate proliferation, transcription, and apoptosis of HSCs. Furthermore, Kim D. C. et al. (2013) also suggested that 5-HT2A receptor antagonists inhibit HSC activation and promote apoptosis. Subsequent studies have shown that serotonin receptors are upregulated in activated HSCs, and 5-HT2B antagonism attenuates fibrogenesis and improves liver function in liver disease models (Ebrahimkhani et al., 2011). The 5-HT7 receptor agonist LP-44 reduces carbon tetrachloride-induced damage in Hep3b cells (Polat et al., 2017). Thus, serotonin seems to be involved throughout the entire hepatic fibrosis pathological process and outcome. However, just as it plays a two-way role in intestinal inflammation, the influence of serotonin on hepatic fibrosis is bifacial. 5-HT exerts differential effects in liver fibrosis due to acting on different receptors, perhaps because different receptors stimulate different intracellular signaling pathways, resulting in convergent biological effects. More detailed research is urgently needed on these speculations concerning the receptor pathway to produce more specific agonists and inhibitors for different receptors, which are expected to play a role in the treatment of liver fibrosis.

In addition to being a neurotransmitter and vasoactive molecule, 5-HT also serves as a mitogen in hepatocytes. Recent studies have shown that serotonin promotes the growth and proliferation of liver tumors, but the specific molecular mechanism whereby this occurs remains unclear. Soll et al. (2012) found that 5-HT receptors 1B and 2B are expressed in 32 and 35% of hepatocellular carcinoma (HCC), respectively, both of which are associated with increased HCC cell proliferation index. Soll’ team found that using serotonin antagonists of receptors 2B reduce the viability and proliferation of Huh7 and HepG2 cell lines. Serotonin may exert a cancer promoting effect in HCC via activation of 5-HT2B receptors (Soll et al., 2010). However, how serotonin works in the development of liver cancer is still unknown. Another study clarified that, in the human HCC cell line Huh7, serotonin stimulates proliferation in serum deprived medium via upregulation and phosphorylation of forkhead transcription factor o subfamily member 3a (FOXO3a), and this effect involved in the 5-HT receptor 2B (5-HT2B) (Liang et al., 2013). Survival analysis showed that elevated levels of 5-HT receptor 1D (5-HT1D) predict a high recurrence rate and a decrease in overall survival in HCC patients. The study found that 5-HT1D aggravates HCC progression through FoxO6 in AKT-dependent and independent manners, revealing the potent carcinogenic effect of 5-HT1D on HCC (Zuo et al., 2019). Moreover, previous studies have shown that 5-HT likely affects hepatoma cells by inducing autophagy. Soll et al. (2010) simultaneously demonstrated that liver biopsy reveals that expression of the serotonin receptor HTR2B is associated with downstream signaling, such as phosphorylation of p70S6K and promotion of proliferation. Activation of the downstream target of mTOR provides evidence that serotonin is involved in the growth of HCC (Soll et al., 2010). The antidepressant indatraline is known to act as a non-selective monoamine transporter inhibitor that blocks neurotransmitter reuptake (including DA, 5-HT, and NE). Indatraline has been reported to increase the number of EGFP-LC3 cells, which express autophagosomes in the cytoplasm (Cho et al., 2016). This phenomenon likely suggests that 5-HT induces autophagy. Recently, Niture et al. (2018) found that serotonin increases the expression of autophagy biomarkers, enhances hepatocarcinomatous cell proliferation, and activates Notch signaling to promote hepatocarcinogenesis. This discovery strongly verifies that 5-HT does indeed act through the autophagy pathway to affect the growth of liver cancer cells. A growing line of evidence indicates that the effect of autophagy on liver cancer is difficult to determine with respect to absolute promotion or inhibition. It is indispensable in the cell physiological and pathological mechanisms, and plays different roles depending on the distinct activation of the pathway. Although our knowledge about the roles of 5-HT in tumorigenesis is still in early stages, the role of 5-HT signaling in promoting HCC progression connecting by autophagy may represent a novel preventive/therapeutic target for hepatic carcinoma with potentially extensive clinical significance.

## Catecholamines and Its Receptor

**FIGURE 2:**
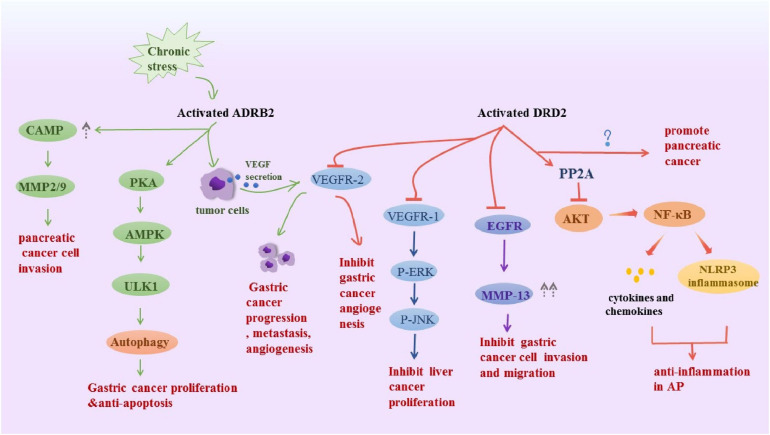
Schematic representation summarizes the DRD2 and ADRB2 signaling in digestive disease. Activated ADRB2 induced pancreatic cancer cell invasion by accumulation of cAMP, promoted gastric cancer proliferation and anti-apoptosis through inducing autophagy. Also, it promoted gastric cancer progression metastasis, angiogenesis via VEGFR-2. The DRD2 signaling activates PP2A and inhibited the phosphorylation of Akt and NF-kappa B to control inflammation, However, it promoted pancreatic cancer, the underlying mechanism is unknown. Besides, it inhibits tumor angiogenesis by inhibiting VEGFR-2 phosphorylation in gastric cancer endothelial cells. DRD2 activation suppresses gastric cancer cell invasion and migration via inhibition of EGFR/AKT/MMP-13 pathway. Moreover, it downregulated VEGFR1, p-ERK and pJNK to ameliorate liver cancer progression.

All catecholamines are derived from L-tyrosine that is converted into levodopa via tyrosine hydroxylase, which is the rate-limiting enzyme in the overall synthesis of catecholamines (Waloen et al., 2017). L-dopa is further manipulated into dopamine in the cytoplasm through the enzyme dopa decarboxylase and the cofactor pyridoxal phosphate (Flatmark, 2000). In peripheral tissues, dopamine B-hydroxylase assists ascorbic acid and oxygen, further manipulating dopamine to form norepinephrine, then through phenyl ethanolamine *N*-methyltransferase and cofactor *S*-adenosylmethionine, ultimately forms adrenaline. DA, NE, and E are classified as catecholamines, and each has specific properties and functions in various organ systems. The dopamine receptor family contains five members that, according to structural and pharmacological similarities, are divided into two subfamilies: the D1-like family, comprising D1 and D5 receptors; and the D2-like family, which includes D2 and D3. Activation of D1-like receptors promotes the accumulation of cAMP in cells, while activation of D2-like receptors inhibits intracellular cAMP levels (Beaulieu and Gainetdinov, 2011), which may be the molecular basis for DA to exert excitatory or inhibitory effects. We have highlighted several non-conventional physiological actions of DA in peripheral systems, including the gut, that go beyond its well-known actions related to gastrointestinal motility and secretion. NE and E act through α (α1 and α 2) and β (β1 and β 2) adrenoceptors in target cells. α1 adrenoceptor mediates its functions by increasing the intracellular calcium level and α2 adrenoceptor downregulates adenylate cyclase and thus inhibits intracellular cyclic AMP. β1 and β2 adrenoceptors activate adenylate cyclase to increase intracellular cAMP (Robison et al., 1967; Thaker et al., 2007). Catecholamines signaling plays a key role in digestive diseases (Figure 2).

### The Protective Effect of DA in Gastrointestinal Diseases

Previous studies have shown that DA regulates the gastrointestinal mucosal barrier. Many studies have shown that dopamine receptors are widely distributed in the gastrointestinal tract and dopamine regulates the gastrointestinal tract function on the movement, secretion, and gastric mucosal blood flow (Li et al., 2006, 2019). Dopamine is currently one of the protective factors involved in the gastrointestinal mucosa. In the stomach, the five subtypes of dopamine receptors have distinct distributions and are primarily localized in the mucosal tissue, along with the muscular layer of the mucosa. The transcripts encoding D1–D3 and D5 are found in dissected muscle and myenteric plexus of the rat intestine, while the mucosa contain D1 and D3–D5 receptors (Li et al., 2006). Subsequent studies have shown that combination of DA and its receptor can resist gastrointestinal mucosal damage, likely owing to several factors as follows: (1) DA reduces gastric tension, intragastric pressure, and staged contractions (Anselmi et al., 2017); (2) DA receptor agonists inhibit gastric acid secretion (Eliassi et al., 2008); (3) DA increases blood flow of the gastric mucosa, improving gastrointestinal mucosal blood circulation (Hiltebrand et al., 2004). Further evidence supports these findings. Holzer and Painsipp (2001) infused rats with drugs such as DA, dobutamine, doxorubicin, and clonidine. Their results showed that the DA group enhances expansion of gastric mucosal vessels and eventually increased gastric mucosal blood. Some scholars have confirmed that the D1 receptor agonist, A 68930, reduces the incidence of gastric and duodenal ulcers in experimental rats, and inhibited the gastric H (+) K (+)-ATPase activity in stress-induced gastric pathology (Rasheed et al., 2010). Therefore, these studies suggested that the mechanism of dopamine-regulated pathways may represent a new treatment for upper and lower digestive tract ulcers.

In the past, the multiple potential anti-inflammatory effects of dopamine have been identified in many other systems (Hanusch et al., 2008; Feketeova et al., 2018). *In vitro*, dopamine has been found to has exert anti-inflammatory effects by suppressing of NOD-, LRR- and pyrin domain-containing 3 (NLRP3) inflammasome in mouse microglia cells and astrocytes (Yan et al., 2015). Xue et al. (2018) observed that a D1-like receptor agonist inhibit IL4 and Interferon γ (IFN-γ) production in Invariant Natural Killer T Cell (iNKT) and suppress iNKT cell-mediated hepatitis in mice, the suppressive effect of dopamine on iNKT cells is mediated by D1-like receptor-PKA pathway. In the same year, dopamine is found to alleviate acute liver injury in mice, and suppress production of TNF-alpha, phosphorylation of c-jun-N-terminal kinase (JNK) induced by lipopolysaccharide (Zhou et al., 2018). However, except for Han’s team, there are few relevant studies focusing on whether dopamine signaling has a similar effect on other organ pathology exploring its underling molecular mechanism. Based extensively on the effects of dopamine on inflammation in other systems, its effects on digestive system have a great deal of potential value. Collectively, the presence of anti-inflammatory targets makes the development and testing of DA agonists an interesting opportunity for potential treatment of inflammatory-related disease.

Additionally, studies have shown that DA is also involved in the pathogenesis of gastrointestinal tumors. It may represent a promising cancer targets in the digestive system. During early time stages of disease, colon cancer patients exhibit lower dopamine compared to normal tissues (Basu and Dasgupta, 1999). Tissue samples in both human and rat gastric cancer present reduced or even absence of dopamine (Chakroborty et al., 2004). The following discoveries have greatly broadened our understanding on the roles of the dopamine receptor in the pathogenesis of digestive system tumors. Chakroborty et al. (2004) proved that a low non-toxic pharmacological dose of DA significantly retards tumor angiogenesis by inhibiting vascular endothelial growth factor receptor 2 (VEGFR-2) phosphorylation in gastric tumor endothelial cells, which express D2 receptors. Ganguly et al. (2010) found that dopamine inhibits insulin-like growth factor-I induced gastric cancer cell proliferation for upregulation of insulin-like growth factor-I receptor via activation of D2 receptors. Huang et al. (2016) showed that DA treatment, acting via D2 receptors, suppresses gastric cancer cell invasion and migration via inhibition of the epidermal growth factor (EGFR)/AKT/MMP-13 pathway and suppression of pituitary tumors via the Rho/ROCK/LIMK signaling pathway. It has also been reported that the D2 receptor antagonistthioridazine reduces the survival rate of gastric cancer cells, induces apoptosis of gastric cancer cells, and plays an important role in the prognosis of gastric cancer cells (Mu et al., 2017). These *in vitro* and animal studies showed that dopamine exerts an important regulatory effect on gastrointestinal diseases via activation of dopamine D2 receptor (DRD2). Treatment with dopamine is not feasible because of severe cardiovascular toxicity. Therefore clinical intervention studies with DRD2 agonists are attractive, especially as these agents are already being used in the clinic for other indications such as Parkinson’s disease and hyperprolactinemia (Beaulieu and Gainetdinov, 2011).

### Bi-Directional Influence of DA in Pancreatic Diseases

Current research on dopamine’s effect on the pancreas is not extensive and profound enough. Han et al. (2017) found that D2 receptors control pancreatic inflammation in acute pancreatitis (AP) by inhibiting NF-κB activation via a protein phosphatase 2A(PP2A)-dependent Akt signaling. Subsequently, Han’s team showed that D2 receptor activation inhibits M1 macrophage polarization, oxidative stress-induced NF-κB and NLRP3 inflammasome activation, suggesting that D2 receptor activation might serve as therapeutic target in AP (Han et al., 2020). Studies have confirmed that dopamine receptor D2 is expressed in both normal pancreatic ductal cells and pancreatic ductal adenocarcinoma cells. And expression of dopamine receptor D2 is significantly increased in human pancreatic ductal adenocarcinoma. Inhibition of this receptor reduces the growth of mouse tumors (Jandaghi et al., 2016). It seems that inhibiting DRD2 provides a targeted approach to pancreatic cancer, and they found that effect may be involved in activating the endoplasmic reticulum (ER) stress.

### DA Servers as a Negative Regulator in Liver Diseases

The latest discoveries have greatly broadened our understanding on the role of the dopamine receptor in liver tumors. On the one hand, thioridazine, a dopamine receptor antagonist, has been shown to induce cancer stem cell differentiation in breast and lung cancer (Yin et al., 2015; Shen et al., 2017). Thioridazine reduces cell viability of HCC cell lines by inducing G0/G1 cell cycle arrest and inhibiting stemness genes CD133 and OCT4 by inhibiting epithelial-mesenchymal transition (EMT)-related genes, such as twist2 and *E*-calcium Mucin, finally inhibiting migration of cancer cells (Li et al., 2015). This suggests that dopamine may be involved in the proliferation of liver cancer cells. On the other hand, dopamine may also exert an anti-cancer effect in HCC via activation of D2 receptors. After stimulation of hepatic cells with fisetin, TGF-β1 secretion is inhibited, and EMT is significantly reduced. Mechanistic studies suggested that fisetin not only downregulates VEGFR1, p-ERK1/2, p38 and pJNK signaling pathways to hinder the progression of liver cancer, but also induces apoptosis of liver cancer cells by activating caspase-3 (Liu et al., 2017). Fisetin, a DRD2 agonist, indicated that dopamine may be of importance in liver cancer progression. These research teams took two different approaches to reach the same conclusion that the growth and migration of cancer cell is hampered via activation or inhibition of different dopamine receptors. Unfortunately, the specific DA regulatory pathways affecting liver cancer behavior are still unclear. We postulate that this may be due to the diversity of dopamine receptors expressing on liver cancer cells; therefore, these agonists and inhibitors exert the same effect because of the agonistic effect or inhibition of different receptors. However, the mechanisms and the importance of DA signaling in HCC cell survival, invasion, and migration remains to be examined in more detail.

### NE/E Mediates a Pro-inflammation Status of Gastrointestinal Diseases

Postganglionic sympathetic neurons innervate lymphoid tissues and immune cells in the gastrointestinal tract. The underlying mechanism may be some new resolution for digestive diseases, as both α and β class adrenergic receptors (ARs) can be expressed by innate immune cells (Cervi et al., 2014). Binding of NE/E to the receptor modulates immune-related cells, thereby affecting gastrointestinal inflammation (Lomax et al., 2009). Tyrosine hydroxylase and dopamine *b*-hydroxylase, two rate-limiting enzymes in catecholamine synthesis, are induced in lamina propria mononuclear cells of the inflamed colon, which is evidence of catecholamine synthesis during colitis (Bai et al., 2009). Some studies suggested that noradrenaline mediates stimulation of the immune response by influencing immune cell migration (Sternberg, 2006). Binding of NE/E to the receptor modulates immune-related cells to upregulate multiple inflammatory cytokines, thereby affecting gastrointestinal and hepatic inflammation (Yang et al., 2000; Lomax et al., 2009). Recently, one study demonstrates that NE blocks secretion of a variety of proinflammatory cytokines by rapidly inducing IL-10 secretion from innate cells in response to Toll-like receptor (TLR) signals, and using beta2-adrenergic receptor (ADRB2)–/– animals and a β2-agonist. It shows that NE is proven to mediate these effects exclusively through the β2-adrenergic receptor in a dextran sodium sulfate (DSS) model of colitis (Agac et al., 2018). Moreover, the crosstalk between gut bacteria and NE/E in the GI tract is also significant. Alterations in the microbial composition of the gastrointestinal tract are believed to contribute to inflammatory and functional bowel disorders. In the gastrointestinal tract, the quantity of gut microbiota far exceeds the number of intestinal epithelial cells by one order of magnitude (Pacheco and Sperandio, 2009). As early as the 1990s, the impact of NE and E to increase the growth of gram-negative bacteria has formed the basis of a new theory regarding host susceptibility to infectious disease. Lyte and his colleagues observed the ability of NE and E to enhance the growth of ram negative bacteria, such as *Escherichia coli* and *Yersinia enterocolitica*, in the early 1992 (Mark Lyte, 1992). And they found that α and β adrenergic receptors involved in this process in the next year (Mark Lyte, 1993). Then, in attempting to further delineate the mechanisms by which NE may influence bacterial pathogenicity, they found norepinephrine induced growth and expression of virulence associated factors in enterotoxigenic and enterohemorrhagic strains of *Escherichia coli* (Lyte et al., 1997). Besides, norepinephrine has been found to supply iron for bacterial growth in the presence of transferrin or lactoferrin. One study 10 years ago reported that norepinephrine is related to *Helicobacter pylori* for the first time. They found both epinephrine and norepinephrine enhance *Helicobacter pylori* growth, with norepinephrine being more effective than epinephrine (Doherty et al., 2009). When *C. jejuni* is grown in iron-limited media in the presence of NE, growth rate, motility and invasion of cultured epithelial cells are increased compared to cultures grown in the absence of NE (Cogan et al., 2007). NE/E obviously helps many kinds of bacteria to invade the stomach and gut. Additionally, communication between the host and the microbiome is not one direction, with hormones being sensed by microorganisms in human gut (Lopes and Sourjik, 2018). A previous study found that bacterial *Citrobacter rodentium* express adrenergic sensors to fully activate its virulence program to successfully colonize its murine host (Moreira et al., 2016).

The effects of the adrenergic system on energy metabolism and the immune system have been shown to modulate cancer metastasis (Kuol et al., 2018). Epidemiological data showed that chronic stress in a negative social and psychological state has an adverse effect on cancer incidence and progression (Chang et al., 2019). NE and E, catecholamine hormones are the primary mediators of chronic stress-induced cancer and are involved in the progression of many cancer cells, including gastric adenocarcinoma (Thaker et al., 2006). Laboratory studies have demonstrated that catecholamines released from the hypothalamic-pituitary-adrenal axis in response to stressors not only affect cellular immunity but also contribute to tumor proliferation, metastasis and angiogenesis through various signaling pathways (Armaiz-Pena et al., 2013; Hassan et al., 2013; Moretti et al., 2013; Shan et al., 2014). Recently, Lu et al. (2017) used a β2-adrenergic receptor (β2-AR) agonist to imitate a stress signal and demonstrated that β2-adrenergic receptor signal enhance angiogenesis by activating VEGFR2 signaling pathway in gastric cancer (GC). Furthermore, stress hormone-induced activation of the ADRB2 signaling pathway plays a crucial role in GC progression and metastasis (Zhang et al., 2019). These findings indicated that ADRB2 signaling regulates GC progression and suggested β2 blockade as a novel strategy to complement existing therapies for GC. Moreover, EMT is responsible for key events in gastric cancer-cell invasion and metastasis (Katoh, 2005). The hypothesis that NE promotes cancer is partly due to its ability to induce EMT procedures and has not been confirmed. Shan et al. found that NE not only significantly induces EMT to alter the morphological characteristics of the stomach but also increases markers for EMT and vimentin expression. Decreased expression of *E*-cadherin, further contributes to cell movement and invasiveness. Their work has shown that NE induces EMT in gastric adenocarcinoma by modulating β2-adrenergic receptor-hypoxia-inducible factor-1α-Snail activity (Shan et al., 2014). Recently, Zhi et al. (2019) demonstrated that induction of autophagy is a novel consequence of β2-adrenergic activation in GC cells. Upon activation of cAMP response element binding protein, chronic stress promotes autophagic flux through the adenosine 5′monophosphate activated protein kinase unc51 like autophagy activating kinase 1 pathway.

### NE/E Aggravates Pancreatic Diseases

Pancreatic cancer has a poor prognosis and is associated with high levels of psychological stress that may adversely affect clinical outcomes (Zabora et al., 2001; Schuller et al., 2012). However, the potential influence of neuropsychological factors on pancreatic cancer has not been investigated to date. Kim-Fuchs et al. (2014) found that beta-adrenergic signaling accelerates pancreatic cancer growth and invasion in the pancreatic microenvironment. Partecke et al. (2016) also found that chronic stress promotes tumor growth and reduced survival of pancreatic cancer patients via beta-adrenergic receptors of tumor cells. Moreover, Pu et al. (2017) found that adrenaline promotes pancreatic PANC-1 cell migration in a dose-dependent manner, inducing a cytoplasmic translocation of RNA binding protein HuR, which in turn activated TGFbeta. Under normoxic conditions, activation of beta-AR receptor transactivates epidermal growth factor receptor (EGFR), which elicits Akt and ERK1/2 in a PKA-dependent manner, leading to accumulation of hypoxia-inducible factor-1 (HIF-1) alpha, and then upregulates expression of its target genes in pancreatic cancer cells (Hu et al., 2010). Therefore, adrenoceptor antagonist appears to be a putative novel treatment for pancreatic cancer.

### Dysfuntion of NE/E Signaling Mediates the Pathology of Liver Diseases

Hepatic fibrosis is characterized by excessive deposition of ECM proteins, with type I collagen predominating. HSCs are the major cellular source of matrix protein-secreting myofibroblasts and the major driver of liver fibrogenesis. “Communication” between the sympathetic nervous system and HSCs are involved in the progress of liver fibrosis. As early as 2003, the Oben team identified the importance of sympathetic nervous system neurotransmitters in liver fibrosis. When fed a hepatotoxic diet, dopamine β-hydroxylase deficient mice lacking NE cannot accumulate activated HSCs and fibrosis is impaired unless treated with an adrenergic agonist (Oben et al., 2003). Soon after, HSCs were found to express adrenergic receptors, release NE and inhibit growth by α and β-adrenergic receptor antagonists. Moreover, the growth of HSCs that do not produce NE is inhibited in dopamine β-hydroxylase-deficient mice, whereas the addition of NE reverses this phenomenon (Oben et al., 2004). Based on their findings, Oben and colleagues concluded that hepatic fibrogenesis requires sympathetic neurotransmitters. However, the mechanisms involved remain unclear. Increasingly, it is becoming evident that catecholamines are actively involved in the production of inflammatory cytokines. Various *in vitro* and *in vivo* experiments have demonstrated that intestinal release of catecholamines stimulates the production of inflammatory cytokines (Yang et al., 2000). NE is found to promote the secretion of inflammatory chemokines (RANTES and interleukin-8), and prazosin (α1 receptor blocker) blocks NE-induced chemokine secretion (Sancho-Bru et al., 2006), indicating that NE has a pro-inflammatory effect that is closely related to liver fibrosis. A pro-inflammatory pattern similar to LPS is observed in NE/E (Aninat et al., 2008). Yang et al. (2001) also found that NE induces hepatocyte dysfunction and elevates plasma TNF-α levels through activation of α2-adrenergic receptors, suggesting that NE induces liver damage at least in part by upregulating TNF-α. Targher et al. (2013) again demonstrated that activated HSCs express functional α/β-adrenergic receptors, which are upregulated in non-alcoholic fatty liver disease patients’ liver with cirrhosis, and they found that this is mediated by NE via p38MAP, PI3K and MEK signaling. Then, Liu’s team discovered that NE promotes HSC-T6 cell activation and secretion of ECM *in vitro* by activating Gα-coupled α1B-AR and α1D-AR and PKC-PI3K-AKT signaling pathways (Liu et al., 2014).

Dysregulation of autophagy has been associated with several human disorders, including metabolic diseases and cancer (Mizushima and Komatsu, 2011). Increasing evidence has demonstrated that autophagy is a key component of the stress response in cancer cells (Lizaso et al., 2013), and G protein-coupled receptors, including the β-adrenergic receptor, can regulate autophagy (Wauson et al., 2014). In mouse liver, the long acting β2-agonist clenbuterol increases autophagosome number in HepG2, and treatment with clenbuterol induces autophagic flux as it decreased levels of SQSTM1/p62 and increased levels of in LC3-II (Farah et al., 2014). In addition, in another study, autophagic regulation by ADRB2 is assessed by immunoblotting, immunofluorescence and immunoprecipitation assays, demonstrating that ADRB2 signaling negatively regulates autophagy by disrupting the Beclin1/VPS34/Atg14 complex in an Akt-dependent manner, reprogramming HCC cells glucose metabolism (Wu et al., 2016). Therefore, adrenoceptor antagonism appears to be a putative novel treatment for HCC. In a follow-up survey, patients with cirrhosis who used β-blockers has a lower risk of developing liver cancer (Herrera et al., 2016). In recent years, it has been found that activation of α1-adrenergic receptors of Küpffer cells promotes the release of inflammatory factors, such as TNF-α, and expedites the development of liver cancer (Huan et al., 2017). Studies have shown that β2-adrenoceptors are upregulated in human HCC (Kassahun et al., 2012). Previous reports have indicated beta-adrenalin enhances cancer cell proliferation (Coelho et al., 2017). In the liver, isoproterenol promotes the growth of hepatoma cell lines HepG2 and MHCC97H (Yuan, 2009), and the α1-AR agonist phenylephrine PE increases stat3 phosphorylation levels in human hepatoma cells and increases DNA transcriptional activity (Han et al., 2008). Collectively, these investigations indicate that NE is a key regulator of hepatoma cells generation and maintenance, but it remains to be determined whether NE is a causative factor of cancer.

## Glutamate and Its Receptors

Glutamate is the major excitatory molecule existing in both the central nervous system and peripheral organs. These actions are mediated via a large range of ionotropic glutamate receptor (iGluR): *N*-methyl D-aspartate (NMDA), α-amino-3-hydroxy-5-methyl-isoxazoleproprionate (AMPA), kainate; and metabotropic glutamate receptor (mGluR). iGluR are directly coupled to cation channels, and their activation evokes fast synaptic events which may lead to longer-term changes in excitability (Bleakman and Lodge, 1998). All metabotrophic glutamate receptors are excitatory neurotransmitters and there are eight mGluR subtypes divided into three major groups: group I, II, and III mGluR, Group I (mGluR1, mGluR5) increase the excitation, whereas group II and III inhibit the release of neurotransmitters (Meldrum, 2000). Previous studies have mainly focused on the biological effect of glutamate in the brain. Recently, increasing evidence has demonstrated that glutamate also participates in the regulation of physiopathological functions in digestive tissues, where the glutamate/glutamate receptor/glutamate transporter system plays an important role in the pathogenesis of these diseases.

### Glutamate Receptor Signal Involved in GERD

Transient lower esophageal sphincter relaxations (TLESRs) is the major mechanism of GERD (Lehmann, 2008). However, mechanisms underlying transient lower esophageal sphincter relaxation are poorly understood (Lehmann and Branden, 2001). A great number of studies have indicated that mGluR5 antagonists may be novel and efficacious strategies in the management of gastro-esophageal reflux disease. They suggest that endogenous activation of mGluR5 is an important component of the pathway triggering or regulating TLESRs. Selective mGluR5 antagonists has been founded to inhibit TLESR in animals and acid reflux in humans. Frisby et al. (2005) found that the mGluR5 antagonist MPEP inhibits TLESR dose dependently, also significantly reduced reflux episodes and increased basal lower esophageal sphincter pressure. In a dog and mouse model, the selective mGluR5 antagonist mavoglurant (AFQ056) has been found to influence the vagal reflex loop and reduced the number of TLESRs (Wu et al., 2010). Also, Randomized controlled trials confirmed that glutamate receptor signal can relax esophageal sphincter. Rohof et al. (2012) found that the volunteers group using selective mGluR5 antagonists shows smaller reductions in TLESRs and reflux episodes (relative to placebo). And ADX10059, a negative allosteric modulator of mGluR5, decreases reflux episodes in healthy subjects. Moreover, in patients with GERD, inhibition of mGluR5 with ADX10059 monotherapy reduces reflux events and improved symptoms in GERD patients (Zerbib et al., 2011). mGluR5 antagonism potently reduces triggering of TLESRs and gastroesophageal reflux.

### Glutamate Receptors Signal Influences GI Motility and Visceral Hypersensitive

Visceral pain is a major clinical problem, mainly in the form of three major functional gastrointestinal disorders: irritable bowel syndrome (IBS), functional dyspepsia (FD), and non-cardiac chest pain (NCCP) (Grundy et al., 2019). Most of the molecular targets so far pursued in clinical trials have been abandoned, mainly due to limited efficacy or adverse events unrelated to the disorder itself. And in this regard mGluR5 appears one of the best candidates, and may therefore support more than one indication. mGluR5 receptor antagonists have been found to inhibit the visceromotor (VMR) and autonomic responses to colorectal distension (CRD) in conscious rats (Lindstrom et al., 2008). An action at peripheral sites mediating the analgesic effects is considered a possibility. Moreover, oral L-arginine L-glutamate ArgGlu (10–30 mg/kg, p.o.) dose-dependently promoted gastric emptying in rats and enhanced gastric motor function, suggesting that it could be a new oral medicine indicated for treatment of upper GI hypofunction or dysfunction like functional dyspepsia (Ishibashi-Shiraishi et al., 2016). However, a study demonstrated that blocking mGluR5 relieves chronic stress related colonic inflammation (Peterlik et al., 2017a). In addition, increasing evidence has indicated that mGluR7 is an important target for reducing anxiety and stress-associated behaviors, and mood disorders are frequently associated with GI dysfunction (Mayer et al., 2001); however, the role of mGluR7 in GI system is currently unknown. Therefore, the present study aimed to evaluate the possible effects of mGluR7 on the visceral hypersensitivity of GI. Julio-Pieper et al. (2010) found that mGlu7 receptor mRNA and protein were highly expressed in mouse colon mucosa and activating mGlu7 receptors modulated fecal water content and strongly induced calcium signaling, further to regulate colonic electrolyte transport. mGlu7 ablation also ameliorated chronic subordinate colony-induced colonic inflammation (Peterlik et al., 2017b). Activation of mGluR7 may attenuate CRD-induced visceral hypersensitivity in experimental IBS and reduce the abnormal immune cytokine response in rats (Shao et al., 2019). In addition, in gastrointestinal tracts malignant tissues, mGluR4 expression was frequently identified in colorectal carcinoma (68%), and expression of mGluR4 was detected in 131 (54%) of 241 colorectal carcinomas and 12 (5%) cases among them showed overexpression in their cytoplasms (Chang et al., 2005). mGluR4 signaling may involve in colorectal carcinomas and that overexpression of mGluR4 is associated with poor prognosis.

Besides mGluR glutamate receptors of the AMPA type, but not kainate receptors, has been found to enhance the efficiency of peristalsis in the guinea-pig colon (Giaroni et al., 2000). NMDA receptor antagonist, memantine, attenuated the body weight loss, colon weight, the plasma levels of interleukin-1β (IL-1β), interleukin-6 (IL-6) (Motaghi et al., 2016). And another antagonist has been found to suppress colon motility and inflammation (Erces et al., 2012). These findings suggested that NMDA antagonist may provide a novel venue for the development of strategies for the treatment of ulcerative colitis. Furthermore, strong evidence demonstrated that activation of peripheral NMDA receptors in colonic tissue sections caused Ca2+-dependent release of the proinflammatory neuropeptides, calcitonin gene-related peptide and substance P (McRoberts et al., 2001). And enhanced activities of NMDA receptors proved to be the underlying mechanism of visceral pain responses in viscerally hypersensitive rats (Cao et al., 2008; Fan et al., 2009). Moreover, Willert et al. (2004) found that NMDA receptor mediated the development and maintenance of human visceral hypersensitivity. Peripheral NMDA receptors are important in normal visceral pain transmission, and may provide a novel mechanism for development of peripheral sensitization and visceral hyperalgesia.

Research has found that glutamate antagonists inhibit proliferation of colon adenocarcinoma and the antiproliferative effect of glutamate antagonists was Ca2 + dependent and resulted from decreased cell division and increased cell death (Rzeski et al., 2001). This results also confirms once again that the increase of intracellular calcium level may be a potential key mechanism for the effects of glutamate and its receptors on digestive tract diseases. It is this excessive intracellular calcium permeation through NMDA channels, which make NMDA channels might thereby regulate cell survival and death pathways during development of gastric cancers (Seo et al., 2011).

### Glutamate Receptors Promote Pancreatic Cancer

Abundant findings suggested that glutamate receptors participate in the progression of pancreatic cancer. The expression of Glutamate receptor GRIA3 was evaluated in human pancreatic cancer tissues. Ripka et al. (2010) found that knock-down of GRIA3 significantly reduced proliferation and migration and enhanced apoptosis. In contrast, overexpression of GRIA3 significantly reduced apoptosis and enhanced both proliferation and tumor cell migration. GRIA3 could be confirmed as a downstream effector of CUX1, which regulated a complex transcriptional program mediating tumor progression (Ripka et al., 2010). Moreover, NMDA receptors signaling controlled invasion of pancreatic neuroendocrine tumor (Li et al., 2018). Treatment of a tumor-derived cell line with NMDA receptors antagonists impaired proliferation and invasion of pancreatic neuroendocrine tumor cell (Li and Hanahan, 2013). In addition, glutamate was found to increase pancreatic cancer cell invasion and migration via activating AMPA receptor activation and Kras-MAPK signaling (Herner et al., 2011).

### Glutamate Receptors Participate in Liver Diseases

Many years ago, Pande et al. (2014) found that mGluR3 up-regulated in rat fibrosis and cirrhosis model. The last study found that alcohol induced the selective expression of mGluR5 in HSCs where mGluR5 activation stimulated 2-arachidonoylglycerol (2-AG) production, and inhibition of mGluR5 attenuated alcoholic steatosis in mice via the suppression of 2-AG production and subsequent CB1R-mediated *de novo* lipogenesis (Choi et al., 2019). In addition, selective blockade of the mGluR5, 2-Methyl-6- (phenylethynyl) pyridine (MPEP), improved hypoxic hepatocyte viability. Significantly, MPEP protected mouse livers in two different vivo models of ischemia reperfusion injury, suggesting its possible protective deployment in the treatment of hepatic inflammatory conditions (Ferrigno et al., 2018).

Recently, mGluR have been identified in peripheral tissues, and aberrant expression or inhibition of the receptor functions in the development of certain cancers. However, the correlation of mGluR activity with HCC remains unknown. Wu et al. (2012) found that inhibiting the activity of mGlu5 has the molecular potential to suppress hepatocarcinogenesis by blocking ERK phosphorylation. In addition, NMDA receptor was proved to present on Kupffer cells, and their activation on primary mouse and human cells limited inflammasome activation by downregulating pyrin domain containing 3 and procaspase-1. This effect may via a β-arrestin-2 NF-kβ and JNK pathway and not via Ca^2+^ mobilization (Farooq et al., 2014).

## Neurotransmitters Signaling Along the Microbiota-Gut-Brain Axis in GI Disease

### The Effects of Various Neurotransmitters on Intestinal Microbes

The intestine is a complex ecosystem harboring a dense and diverse microbial community called the gut microbiota, which co-evolved with the host to develop a mutualistic relationship. The gut microbiota is considered a virtual endocrine organ, producing molecules that are able to interact with the host physiology and trigger responses at the local and distant levels (Zhang and Davies, 2016). Any perturbation in host–microbiota crosstalk can be an initiating or reinforcing factor in disease pathogenesis. The gut-brain axis is a bidirectional communication system between the central nervous system and the gastrointestinal tract, in which neurotransmitter play as a key medium in this communication. Most of previous studies showed the key role of gut microbiota on metabolism of neurotransmitters and GI disease (Agus et al., 2018; De Vadder et al., 2018). In this part, we intend to focuses on the effects of various neurotransmitters on intestinal microbes to facilitate a better understanding of the pathogenesis of human digestive diseases.

A last study demonstrated that elevating levels of intestinal lumenal 5-HT by oral supplementation or genetic deficiency in the host 5-HT transporter increased the relative abundance of spore-forming members of the gut microbiota (Agus et al., 2018). 5-HT promotes their fitness in the intestine. Kwon et al. (2019) found that 5-HT directly stimulated and inhibited the growth of commensal bacteria *in vitro*, exhibiting a concentration-dependent and species-specific effect. 5-HT also inhibited β-defensin production by HT-29 colonic epithelial cells (Kwon et al., 2019). These findings support the emerging concept that bidirectional signaling pathways can influence bacterial community structure and exert effects on host physiology. Emerging evidence suggests that the diversion of the tryptophan metabolism from the 5-HT pathway toward the Kynurenine (Kyn) pathway may have an important role in the manifestation of psychiatric disorders such as anxiety and major depression (Kennedy et al., 2017). In the use of germ-free (GF) mice, induction of depressive mood after fecal transplantation was associated with an increase in the Kyn/tryptophan ratio (Carabotti et al., 2015). Changes in tryptophan metabolism have been correlated with the manifestation of depressive symptoms also in IBS patients (Keszthelyi et al., 2013). This could open a therapeutic opportunity for adjuvant treatment of some IBD and IBS symptoms associated with changes in the levels of Kyn pathway metabolites along the brain-gut axis.

The intestinal epithelium is a critical barrier between the internal and external milieux of the mammalian host. Neurotransmitters appear to influence epithelial associations with bacteria in the intestinal lumen. Both NE and DA have been shown to alter the mucosal attachment or invasiveness of bacterial pathogens such as *enterohemorrhagic Escherichia coli* (EHEC) or serovars of *Salmonella enterica* by acting on the intestinal mucosa (Chen et al., 2003). The serosal application of NE produced an increase in *luminal S. enterica* serovar Choleraesuis and EHEC internalization in porcine Peyer’s patch explants. This effect was abolished in tissues pretreated with the alpha -AR antagonist phentolamine (Green et al., 2003; Chen et al., 2006). Besides, *Escherichia coli O157:H7* possesses a receptor for host-derived epinephrine/norepinephrine that can be blocked specifically by adrenergic antagonists (Clarke et al., 2006; Meng et al., 2016). Based on the information available, many more questions can be asked. For example, does endogenous NE (and potentially DA) act to regulate aspects of bacterial sampling at mucosal immune recognition and processing sites, as in intestinal Peyer’s patches? The relationship between sympathetic activity with mucosal immunity and inflammation, bacterial colonization, or the risk of mucosal infection may offer fruitful areas for investigation.

### Microbial Production of Neurotransmitters Play a Role in GI Disease

A bi-directional cross-talk between microbiota and the endocrine system is emerging with bacteria being able to produce hormones (e.g., serotonin and dopamine). Serotonin (5-HT) is a key regulator of GI motility and secretion. Recent studies highlight a role for the microbiota in regulating blood 5-HT levels, in germ free animals, there is a significant reduction of serotonin in the blood and colon of mice compared to conventionally colonized controls (William et al., 2009; Sjögren et al., 2012). Besides, intestinal ECs are morphologically larger in the former rats (Uribe et al., 1994), which suggests that microbes could impact the development of 5-HT-producing cells. Interestingly, some species of bacteria grown in culture can produce 5-HT (Tsavkelova et al., 2006), raising the question of whether indigenous members of the microbiota contribute to host 5-HT levels through synthesis. To explore how pathways of 5-HT metabolism are affected by the gut microbiota, Yano et al. found ed that the microbiota promotes 5-HT biosynthesis from colonic ECs. And, colonic PCPA [the Tph inhibitor para-chlorophenylalanine (PCPA)] administration blocks the ability of the microbiota to promote colonic and blood 5-HT suggests that gut microbes require host TPH activity to upregulate peripheral 5-HT (Yano et al., 2015). In addition to EC cells, gut microbes have been found to promote colonic 5-HT production through an effect of short-chain fatty acids on enterochromaffin cells (Reigstad et al., 2014). Therefore, microbiota could influence 5-HT-related GI disease symptoms. While it has not been confirmed that the human microbiota modulates norepinephrine or dopamine *in vivo*, there is accumulating evidence suggesting it may, or at least play a role in host biosynthesis/catabolism. With regards to norepinephrine, a recent study leveraging germ free animals found that mice without bacteria have substantially reduced levels of norepinephrine in the cecal lumen and tissue, and that cecal levels of norepinephrine could be restored via colonization with a microbiota or with a mixture of 46 Clostridia species (Asano et al., 2012). This finding strongly suggests the microbiota influences levels of norepinephrine in the lumen, but whether the bacteria were producing norepinephrine directly or modulating host production was not determined. Besides, Several bacteria have been reported to be able to produce dopamine, such as *Bacillus cereus*, *Bacillus mycoides*, *Escherichia coli* and so on (Tsavkelova et al., 2000). This host-microbiota interaction contributes to a growing appreciation that the microbiota regulates many aspects of GI physiology and Pathophysiology by signaling to host cells.

## Conclusion

In the past few decades, many studies have demonstrated that neurotransmitters regulate the physiological and pathological functions of various tissues and organs. However, there are few reviews that discuss the role of neurotransmitters in digestive tract diseases, which is a meaningful and worth exploring field. Previous studies have confirmed that neurotransmitters play an essential role in maintaining the physiological function of digestive tract organs. The imbalance of neurotransmitter release, excessive activation of receptors, or loss of their function is closely related to the pathological state of digestive tract organs. 5-HT and its receptors are mainly distributed in smooth muscle cells, so 5-HT signaling is very important for the motility balance of the esophagus and gastrointestinal tract. Clinical application of 5-HT receptor agonists and SRIS can improve the symptoms of IBD, IBS, and other dynamic diseases. At the same time, animal experiments also found that the administration of receptor blockers aggravated gastrointestinal inflammation, which seemed to suggest that 5-HT signaling is involved in the defense of gastrointestinal inflammation. However, many studies have found that 5-HT signal maintains or even aggravates intestinal inflammation by activating immune cells to release inflammatory cytokines. Therefore, more research is needed on the role of 5-HT. DA, E and NE are the main mediators of SNS in regulating intestinal inflammation, tumor growth and progression. This text summarizes the different effects of catecholamine. The activation of DA receptors inhibits angiogenesis and stimulates tumor immunity, while NE and E stimulate angiogenesis and inhibit tumor immunity, blocking the signal transduction of adrenergic receptors, which also hinders the occurrence and development of tumors. At the same time, DA also plays a protective role in pancreatitis. More importantly, our team’s research found that dopamine reverses the increase of α-SMA in HSCs stimulated by TGF-β1, and reverses the increase of autophagy induced by TGF-β1 in HSCs. These results suggest that dopamine plays a protective role in liver fibrosis, which may be achieved by affecting the activation of HSCs. The specific mechanism needs further study. As a result of these protective or promotive effects, classic neurotransmitter related drugs, such as β-AR antagonists, serotonin receptor antagonists, AChR antagonists and DA receptors agonists, may have clinical significance in the treatment of gastrointestinal diseases and are expected to become candidates for combined drug therapy. No clinical trials of DRD2 agonists have been found in patients with gastrointestinal cancer. Therefore, the clinical intervention of DRD2 agonists and beta-blockers is of great value and attraction, especially because these drugs are known to be used in the treatment of other indications such as Parkinson’s disease. The majority of preclinical and clinical studies, up to now, have been used iGluR antagonists, whose potential clinical usefulness is, however, limited by the variety of side effects. Other approaches would include the discovery of modulators of the glycine site associated with NMDA receptors, of the reuptake systems, as well as of mGlu receptor allosteric modulators to provide fine tuning of the glutamatergic neurotransmission. An innovative and intriguing approach is represented by the possibility to modulate neurotransmitters signaling along the microbiota-gut-brain axis by influencing the microbiota composition. One of the possible approaches in this field is the use of probiotics, which are beneficial bacteria yielding positive health outcomes. In short, numerous clinical neuroactive drugs, once explored, will greatly help to reduce the pain of gastrointestinal diseases and even cancer patients, which will be of great significance.

## Author Contributions

All authors listed have made a substantial, direct and intellectual contribution to the work, and approved it for publication.

## Conflict of Interest

The authors declare that the research was conducted in the absence of any commercial or financial relationships that could be construed as a potential conflict of interest.
